# Supplementary material for: Pervasive relaxed selection on spermatogenesis genes coincident with the evolution of polygyny in gorillas
Source: eLife. 2026 Jul 7;13:RP94563. doi: 10.7554/eLife.94563 (PMC13341116; doi:10.7554/eLife.94563)
Supplement: Peer review history

# Editor report: eLife Assessment

**DOI:** 10.7554/eLife.94563.3.sa0

**Authors:** Wei Yan

**Affiliations:** Washington State University United States

**Keywords:** Compelling, Important

This **important** study reports that genome-wide signatures of relaxed purifying selection in genes associated with male fertility may reflect an evolutionary response to reduced sperm competition in the gorilla mating system. The authors present **compelling** data that robustly support their central conclusion. This work will be of broad interest to investigators in evolutionary biology and reproductive biology.

# Referee report: Reviewer #1 (Public review)

**DOI:** 10.7554/eLife.94563.3.sa1

This manuscript describes the pattern of relaxed selection observed at spermatogenesis genes in gorillas, presumably due to the low sperm competition associated with single-male polygyny. The analyses to detect patterns of selection are very thorough, as are the follow-up analyses to characterize the function of these genes. Furthermore, the authors take the extra steps of in vivo determination of function with a Drosophila model.

This is an excellent paper. It addresses the interesting phenomenon of relaxation of selection as a genomic signal of reproductive strategies using multiple computational approaches and follow-up analyses by pulling in data from GO, mouse knockouts, human infertility database, and even Drosophila RNAi experiments. I really appreciate the comprehensive and creative approach to analyze and explore the data. As far as I can tell, the analyses were performed soundly and statistics are appropriate. The Introduction and Discussion sections are thoughtful and well-written. I have no major criticisms of the manuscript, just a few minor thoughts.

In the "Caveats and Limitations" section of the Discussion, the first paragraph of this section states the obvious that genetic manipulation of gorillas is not feasible. Beyond a reminder to the reader that this was a rationale for the Drosophila work, it isn't really adding much insight.

I do agree with one of the initial reviewers that a comparative approach would add powerful perspective on the evolution of these genes. At the same time, I agree with the authors that the present work is comprehensive and can stand in its own in providing convincing evidence that many male reproductive genes in gorillas have experienced relaxed selection, without reference to other species with similar mating systems. I do not think that the elephant seal data adds a useful perspective.

# Referee report: Reviewer #3 (Public review)

**DOI:** 10.7554/eLife.94563.3.sa2

In this study the authors tested for alterations in selection intensity across ~13,000 protein coding genes along the gorilla lineage in order to test the hypothesis that the evolution of a polygynous social system resulted in relaxed selective constraint through a reduction in sperm competition. Of these genes, 578 exhibited signatures of relaxed purifying selection that were enriched for functions in male germ cells including meiosis and sperm biology. These genes were also more likely to be expressed in male germ cells and to contain deleterious mutations. Functional analysis of genes not previously implicated in male reproduction identified 41 new genes essential to male fertility in a Drosophila model. Moreover, genes under relaxed selective constraint in the gorilla lineage were more likely to contain loss of function variants in a cohort of infertile men. The authors conclude that their results support the hypothesis that the emergence of a polygynous social system may have reduced the degree of selective pressures exerted through sperm competition.

(1) The identification of novel genes involved in spermatogenesis using signatures of relaxed selective constraint coupled to in vivo RNAi in Drosophila offers a proof of principal as to the power of evolutionarily-informed functional genomics that has been largely underutilized.

(2) The analysis is restricted to protein-coding regions of genes that have single, orthologous sequences spanning 261 mammalian species, and as such is a non-random set of 13,310 genes that have higher evolutionary conservation. While this approach is necessary for the analyses being performed, it excludes non-coding regions, recently duplicated genes/gene families, and rapidly evolving genes, which are all likely subject to stronger selection as compared to evolutionarily conserved genes (and gene regions). Thus, the conclusions of relaxed selective constraint as being pervasive could be missing a large number of the most strongly selected genes, many of which may include sex and reproduction related genes.

(3) The identification of genes showing relaxed selection along the gorilla lineage, which are overrepresented in male reproduction, supports the hypothesis that the emergency of polygyny resulted in relaxed sperm competition and is the driving force behind their observations. To more fully test this hypothesis the authors contrast their findings to observations in elephant seals, however of the 573 genes under relaxed selection in gorillas only 14 show a similar pattern. These genes are not enriched for male reproductive function, and may be under-powered or result from variation in reproductive strategies in gorillas as compared to elephant seals that mate seasonally.

(4) The comparisons of human males with infertility to a large number of healthy males from a separate cohort can lead to genetic differences related to population structure or differences in study recruitment independent of infertility, and care must be taken to avoid confounding. Population structure is more likely to affect patterns of rare variation (including loss of function mutations), even when controls are ascertained using similar enrollment criteria, geographic regions, racial/ethnic and national identities. In this study, the MERGE cohort is largely recruited from Germany, vs. a geographically more broadly recruited control cohort gnomeAD. The authors performed a sub-cohort analysis among individuals identified as having predominantly European genetic ancestry within MERGE, to that of non-Finnish European individuals from genomeAD, and find similar results, thus strengthening their findings.

# Author comment: Author response

**DOI:** 10.7554/eLife.94563.3.sa3

**Authors:** Jacob D Bowman, Neide Silva, Erik Schüftan, Joana M Almeida, Rion Brattig-Correia, Raquel A Oliveira, Frank Tüttelmann, David Enard, Paulo Navarro-Costa, Vincent J Lynch

**Affiliations:** University at Buffalo, State University of New York Buffalo United States; University of Lisbon Lisbon Portugal; University of Münster Münster Germany; University of Lisbon Lisbon Portugal; Instituto Gulbenkian de Ciência Oeiras Portugal; Instituto Gulbenkian de Ciência Oeiras Portugal; University of Münster Münster Germany; University of Arizona Tucson United States; University of Lisbon Lisbon Portugal; University at Buffalo, State University of New York Buffalo United States

The following is the authors’ response to the original reviews.

**Reviewer #1 (Public Review):**

This manuscript describes the pattern of relaxed selection observed at spermatogenesis genes in gorillas, presumably due to the low sperm competition associated with single-male polygyny. The analyses to detect patterns of selection are very thorough, as are the follow-up analyses to characterize the function of these genes. Furthermore, the authors take the extra steps of in vivo determination of function with a Drosophila model.

This is an excellent paper. It addresses the interesting phenomenon of relaxation of selection as a genomic signal of reproductive strategies using multiple computational approaches and follow-up analyses by pulling in data from GO, mouse knockouts, human infertility database, and even Drosophila RNAi experiments. I really appreciate the comprehensive and creative approach to analyze and explore the data. As far as I can tell, the analyses were performed soundly and statistics are appropriate. The Introduction and Discussion sections are thoughtful and well-written. I have no major criticisms of the manuscript.

We thank you for your kind words!

The main area that I would suggest for improvement is in the "Caveats and Limitations" section of the Discussion. Currently, the first paragraph of this section states the obvious that genetic manipulation of gorillas is not feasible. Beyond a reminder to the reader that this was a rationale for the Drosophila work, it isn't really adding much insight. The second paragraph is a brief discussion of the directionality of change. I think it comes across as overly simplistic, with a sort of "well, we can never know" feel. Obviously, there are plenty of researchers who do model change to infer direction and causation, and there are plenty of published papers attempting to do so with respect to mating systems in primates.

We understand these statements might seem trivial, but they are meant to fully acknowledge, particularly to non-evolutionary biologists, the fact that we can’t do the genetics to “prove” these putatively deleterious mutations really are so (hence the statement about forward/reverse genetic experiments), nor causation (since this mating system evolved once in the history of gorillas we cannot know directionality in this lineage, although we could infer it if we had species in which different stages were extant, for example).”

I do not think the authors need to remove these paragraphs, but I do encourage them to turn the "Caveats and Limitations" section into something more meaningful by addressing limitations of the work that was actually done rather than limitations of hypothetical things that were not done. A few areas come to mind. First, the authors should discuss the effect of gene-tree vs species-tree inconsistencies in the analyses, which could affect the identification of gorilla-specific amino acid changes and/or the dN/dS estimates. Incomplete lineage sorting is very common in primates including the gorilla-chimp-human splits (Rivas-González et al. 2023). It would be nice to hear the authors' thoughts on how that might affect their analyses. Second, the dN/dS-based analyses assume the neutrality of synonymous substitutions. Of course, that assumption is not completely true; it might be true enough, and the authors should at least note it as a caveat. Third, and potentially related, is the consideration that these protein-coding genes may be functioning in other ways such as via antisense transcription. The genes under relaxed selection may be on their way to becoming pseudogenes and evolving as such at the sequence level, but many pseudogenes continue to be transcribed sense or anti-sense in a regulatory purpose. I don't think there is a way to incorporate this into the authors' analyses but it would be nice to see it acknowledged as a caveat or limitation.

We thank you for the helpful suggestion and have added a discussion of these issues in the reworked Caveats and limitations section (lines 639 - 710).

**Reviewer #1 (Recommendations for The Authors):**

This is an excellent paper with thorough and creative approaches to address an interesting connection between genotype and phenotype. Stylistically the paper is very well written.

We thank you for your kind words.

Page 3: I suggest deleting the word "vaginal" so the sentence reads "... the evolution of female traits such as anatomical features that allow female control...". Most of the well-documented examples of cryptic female choice are in animals that do not have vaginas like insects, fish, and birds, including the reference given at the end of the sentence (Brennan et al. 2007 on waterfowl).

We agree and have made this edit.

Page 3: I would delete the words "multimale-multifemale" when discussing gorillas, to make the sentence read "Most gorillas, for example, live in groups with age-graded...". The use of "multimale-multifemale" here is not exactly wrong, but can be confusing to the reader since the authors essentially use "multimale-multifemale" as a synonym for "polygamous" in the previous paragraph.

We agree and have made this edit.

The writing in the Materials and Methods fluctuates between present and past tense. The authors should pick a consistent style, probably past tense by convention.

We have edited the Materials and Methods only to use past tense.

"Drosophila" is italicized sometimes, but not sometimes not. Make consistent.

To ensure consistency, italics were used only when genus and species were shown together (i.e., *Drosophila melanogaster*).

In the main text, a few reference typos/confusions:

Box 1, Figure 1B caption: I believe this "Dixson, n.d." reference should be Dixson (2009), if it refers to the book (Oxford Press).

Yes, that is the case. Thank you for having spotted this. The reference has been corrected.

Page 21: The authors use the term "false exons" and "fake exons" in the same paragraph. Are these the same thing? If so, just use "false exons" both times.

These are the same, we have changed fake to false.

Page 22-23, maybe elsewhere: The Smith et al. reference includes Martin's first name.

Thank you for bringing this issue to our attention. The reference has been corrected.

Page 25: in the parenthetical listing of scientific species names, the word "and" should not be italicized. In this same section, there's really no reason to include "gorilla" as the subspecies. It isn't given for the other species.

Corrected.

Page 27: Missing period in the second paragraph after "(Guyonnet et al. 2012)".

Corrected.

Page 29: Should read "... available in gnomAD that would allow us to exclude..." (or possibly "... available in gnomAD that would allow the exclusion of ...").

Corrected.

Page 33, figure legend off Appendix Figure 1A: "gray line" not "gray liner".

Corrected.

Box 1, Figure 1A: This is confusing in a few ways. First, the gorilla red dot is labeled "Gorilla", but the chimpanzee and bonobo dots are not labeled. Perhaps in the legend the colors could be indicated, such as "... percentage of body mass for gorilla (red), common chimpanzee (dark blue), and bonobo (light blue)"? Secondly, the bar chart shows the testes/body mass ratio but it is not clear what they are scaled to. Should there be a second y-axis on the right side of the plot?

The bar chart showed the testis weight/body weight ratio (log), but it is not really necessary. We have removed the bar chart and labeled chimpanzees and gorillas.

Figure 1D: I found myself confused by the vertical label of "Percent of genes with w>1 in Gorilla". Because all genes are in the stacked histogram, my first thought was that ~99% of the genes have w>1 (gray). Would be more clear if the label was the same as 1G ("Percent of genes").

We agree and have made this change.

The text in the figures is extremely small. I don't know what it will look like once it is fully formatted for publication, so I'll leave those concerns to the editor/publisher.

We will wait until the proofs to determine if this figure needs to be split into multiple figures with larger text.

References in the reference section need a LOT of cleaning up. It does not appear that any manual editing was done. Please check for consistency in capitalization, italicization, abbreviations, missing information, etc. The level of neglect to this section is frankly unprofessional.

I (VJL) apologize for this; it is entirely my fault. To explain but not justify, I have dyslexia, and the shifting combination of text, numbers, punctuation, fonts, and font styles makes it difficult to see the inconsistencies. To mitigate this, I use a reference manager to format references (like everyone else) and almost always have someone proofread the reference section, but I didn’t do that with this manuscript. I apologize for the oversight. My dedicated co-authors have cleaned the reference section.

**Reviewer #2 (Public Review):**

As outlined in the public review, this is a nicely executed molecular evolutionary study. The analyses and overall patterns described in gorillas appear rigorous and convincing. The fundamental limitation here is a lack of comparative context to specifically establish the connection to mating system or the uniqueness of these overall patterns to gorillas.

We thank the reviewer for the compliments. However, there is some confusion about the hypothesis we tested. We hypothesized that genes involved in male reproductive biology would have relaxed selective constraints in gorillas because of their mating system, not that polygynous mating systems would lead to relaxed selection. While that may be true, it is not the hypothesis we tested, nor do we state that the overall pattern we observe is unique to gorillas. Our data, however, support our claims: (1) We performed an unbiased selection scan in gorillas and identified genes with K<1, an evolutionary signature of reduced selection intensity; (2) We found that those genes were enriched for male reproductive functions; and (3) Some of those genes had effects on male reproduction in both Drosophila screens and in infertile men. These are the results one would expect if our hypothesis were true.

To partly address the concern that our results do not have a connection to mating systems or may be an overall pattern rather than a gorilla-specific one, we ran RELAX using the same dataset but in the elephant seal, another species with a highly polygynous mating system. Although elephant seals are a polygynous species, they differ from gorillas in that their spermatogenesis does not undergo persistent deterioration, but instead follows a seasonal pattern. According to the comprehensive study by Laws (The Elephant Seal (Mirounga Leonina Linn.): III. The physiology of reproduction; Scientific Reports, 15, Falkland Islands Dependencies Survey, 1956), male gamete production is upregulated during the mating season and is mostly inactive throughout the rest of the year. Of the 573 genes with K<1 in gorillas only 14 also have K<1 in elephant seals, which had 350 genes with K<1. A GO analysis of the 350 elephant seal K<1 genes does not identify enrichment in spermatogenesis-related terms. In fact, the list of GO terms is quite broad. A potential, if admittedly speculative, interpretation of these findings is that although polygynous, the selective pressure on elephant seal spermatogenesis is not relaxed (unlike in gorillas) because of the seasonal nature of their mating period. In other words, by having a temporally narrower window for reproductive success than gorillas, the selective constraint on male gametogenesis in seals is not weakened. Regardless, the low overlap in relaxed genes between the two tested polygynous species support the view that this reproductive strategy is probably associated with different evolutionary signatures in the genome (depending on the species), a likely reflection of the complex, nuanced and multi-factorial aspects of such strategies. We include this analysis in the Appendix (lines 1112 - 1132).

While there is much that I like about the study and approach, this is a substantial shortcoming that really limits the significance of the, especially given that lineage specific patterns were also analyzed by Scally et al. (2012) over a decade ago.

While Scally et al. (2012) reported the initial sequencing, assembly, and analyses of the gorilla genome, the method they used to characterize selective pressure on coding genes - the branch and branch-site model implemented in PAML - is misspecified to detect relaxed selection (PMID: 25540451). Under relaxed selection, the d_N_/d_S_ of sites under purifying selection will move towards 1, the d_N_/d_S_ of sites under positive selection will also move towards 1, and some sites will not experience a change in d_N_/d_S_. The PAML test used Scally et al. (2012) averages d_N_/d_S_ across all sites, rather than having distinct rate categories for each of the three selection classes. A change in d_N_/d_S_ toward 1 under the PAML model can arise because the strength of positive selection is weaker in the foreground lineage than the background lineage, even if there is still positive selection acting on some sites. Averaging across all sites also means there is little power to detect relaxed selection, even if it is relaxed selection. Furthermore, the PAML test used by Scally et al. (2012) is underpowered to detect relaxed selection because it depends on selective regimes in background species. Scally et al. (2012) also used six species, which underpowers their test of relaxation, because if one or more of those species experience an increase in their d_N_/d_S_ rate, the background rate will increase giving the appearance of a decrease in the gorilla lineage even if its d_N_/d_S_ rate has not changed. We elaborate on this in the Appendix section (lines 1036 - 1073). Finally the method implemented in PAML does not allow for synonymous rate variation across sites or multi-nucleotide mutations per codon, ignoring synonymous rate variation dramatically inflates the false positive rates in selection tests (PMID: 32068869) as does ignoring multi-nucleotide mutations (PMID: 29967485 and PMID: 37395787); we have added a discussion of these issues in our Caveats and limitations section (lines 683 - 710).

**Reviewer #2 (Recommendations for The Authors):**

Specific comments

Framing: Overall, the connection between mating system is referred in variable levels of certainty, some appropriate, others overstated. The paper title uses 'coincident' which is appropriate, but also at odds with the stronger conclusions that are emphasized throughout. Elsewhere the phrasing is much stronger (abstract, discussion) implying a direct statistical association with mating system variation that has not been established. Elsewhere the term 'association' is used in the same manner, but in instances where a statistical association is tested and demonstrated (tests of enrichment, etc).

We are unsure why the Reviewer considers our claims overstatements. The patterns of molecular evolution we found are ‘associated,’ and 'coincident with,' and we believe our results are ‘compelling’. Our tests for relaxed and positive selection are statistically associated with a polygynous social system which we a *priori* hypothesized. We have taken care to ensure a more consistent framing of this connection throughout the manuscript to avoid potential misinterpretations of causality.

Page 7, elsewhere- It is essential to compare the reported patterns (percentage of relaxed genes in gorilla, patterns of enrichment, etc) to other primate lineages to identify if this number is enriched due to mating system or if these patterns are unusually for sperm genes across mammals. The implication here and throughout is that the specific pattern reflects specific aspects of gorilla mating biology, but this is never established. Additionally, it would be interesting to know the relative number of genes under positive selection across species (or across great apes).

We agree that if we were using a PAML-like approach that these controls would be informative. But with the RELAX method the foreground K is compared to the background K, K only becomes significantly less than one if there is relaxing in the intensity of selection in the foreground. If these patterns were common to sperm genes across mammals the background and foreground K would not be significantly different. Our a *priori* hypothesis was that genes related to male reproductive biology would show evidence of a decrease in the intensity of selection (both positive and purifying), which we tested and found to be true. In this regard, we can conclude that the gorilla mating system is associated with patterns of molecular evolution in the species’ genome.

While we too would find it interesting to know the relative number of genes under positive selection across species (or across great apes), that is not the study we performed and is beyond the scope of this one (and we only identified 96 genes that were positively selected in gorilla suggesting that few genes are positively selected across species).

Page 8, bottom, elsewhere- "13,491 background set" elsewhere this is 13,310 (abstract). The number of genes here is different, and the set seems to change across multiple parts of the paper without explanation. This could be a simple typo, however, it may affect statistical analysis if the problem is widespread, especially when assessing enrichment of (presumably) small sets of genes.

This is partly true and partly a typo. We generated 13,491 alignments, 13,310 of which had HUGO gene symbols. These 13,310 genes were used in all subsequent studies. We have re-written the text to clarify this point, and have added a statement: “We thus generated a dataset of 13,491 orthologous coding gene alignments from the genomes of 261 Eutherian mammals, corresponding to 62.7% of all protein-coding genes in the gorilla genome. Of the 13,491 alignments, 13,310 had an identifiable HUGO gene symbol and were used in all subsequent analyses (lines 158 - 162).”

Related to this, it is difficult to determine how many genes these GO associations are based on. Even small numbers of genes can result in very significant results with these tests. How many genes are these associations based on? This connection is a key component of the overall narrative that changes in sperm competition have a large effect on genome-wide shifts.

All analyses are based on the 13,310 genes with identifiable HUGO gene symbols, including over-representation analyses (ORA). Our dataset submitted with this manuscript includes these 13,310 genes (as well as the genes with K<1 and K>1). The number of genes used as the foreground is the 578 with K<1, these genes are given in Figure 1 – source data 3. The minimum number of genes annotated in a GO or pathway term was 3. While it is unlikely that statistically significant GO term enrichments result from a few genes annotating to each term, that scenario would produce small P-values, the false discovery rate would be high and readers can decide what false discovery they are willing to accept.

How many of these 578 genes are plausibly related to reproduction? Apologies if I missed this detail, but Figure 3 does not convey this. Could you speak to this directly in the text and include a table or supplemental table of the GO terms to show the differences in enrichment between classes of genes, and counts per term?

These data are included in Figure – 3 source data 1.

One of the key results is the relative frequency of relaxed constraint versus positive selection. This is expected on some level as the form of recurrent positive directional selection detected with these models is usually relatively rare. However, it is not at all clear that it is rarer in gorillas versus other mammals, as implied.

Our comparison of relaxed constraint to positive selection was to explore if more genes experienced one pattern of molecular evolution or the other within gorillas, we do not imply that it is rarer in gorillas than in other mammals.

Likewise, I was wondering how the dataset itself may be biased toward this result. If I understand correctly, you are requiring very high levels of conservation (251/261 genes) for inclusion in the dataset, resulting in ~60% of all gorilla genes being included. Rapidly evolving genes that are targets of recurrent positive selection often also tend not be highly conserved across such a deep phylogenetic sample. It would be good to acknowledge this potential bias when implying meaning to the differences in relative rates of the two forms of selection.

Our results are unlikely to be subject to this bias. The RELAX test relies on accurately estimating K in background lineages, which requires that we include as many species as possible. The tradeoff is a reduction in the number of genes included in the dataset due to evolutionary dynamics across a wide range of species. However, it's not that 40% of the genes are excluded because they are evolving so rapidly we cannot identify or align them, it mainly reflects the fact that we cannot identify the gene in 251 of the 261 species included in the dataset (due to gene loss, etc).

Page 9 - The results here (and in Figure 3D) shows that relaxed genes are enriched broadly across spermatogenesis cell types except for Sertoli cells. But the Sertoli cells and a few non-significant cell types are the only thing to compare to. Instead, it would be interesting to identify single cell expression patterns from other tissues- or even bulk RNA as sc-RNA may be limited in the species. This would show that these genes are enriched in testis compared to other tissues, as opposed to just being broadly expressed. Additionally, the authors could compare to the other primate testis sc-RNA available in Murat et al. Without such comparisons the interpretations here seem limited.

We did not test whether K<1 were enriched in other cell types because: (1) we had an a *priori* hypothesis that genes with K<1 would be enriched in cells involved in male reproduction, rather than enriched in cell types in the testis compared to any other cell type; and (2) The number of genes with K<1 is relatively small and the number of known cell-types in very large, at least one estimate points to ~400 major cell types in a higher primate (PMID: 37722043). Using a P-value of 0.05 from a hypergeometric or Fisher's exact test and a Bonferroni correction to control for multiple hypothesis testing, we would need the P-value for enrichment in any cell type to be 0.000125, which we are unlikely to achieve.

More comprehensive functional comparisons could provide evidence that even though relaxed constraint is present in all lineages, perhaps relaxed constraints in the gorilla lineages are more related to sperm formation and function.

The RELAX test is a relative one; while relaxed constraint may be present in other lineages, to observe a statistically significant K<1 in gorillas the degree of relaxation would have to have a greater effect size in gorilla than in other lineages.

I was also a little unclear what to make of the interpretation of K<1 versus K >1 enrichment by cell type. The enrichment of K<1 is called out as noteworthy because this is when the spermatogenesis specific genes begin to be expressed, but then the K > 1 result is dismissed as occurring during pachytene which is a transcriptional permissive state of testis. To be clear, pachytene is also a critical checkpoint for fertility and enhanced purifying selection at this step could be reasonably interpreted as being at odds with the entire erosion of reproduction argument. This seems to be a selective interpretation for the overall narrative. Also, permissive transcription is not only limited to the pachytene stage and the relaxation of constraint concomitant with increased specificity and permissive expression during the later stages of spermatogenesis is a well-known result in mammals, and not anything that can be ascribed gorillas and their change in mating system.

We agree with the Reviewer’s comment and have removed the K<1 versus K>1 interpretation from the manuscript.

Page 13 - The LOF enrichment identified from this random sampling is borderline significant. An improved approach would be to perform permutations of random samplings and identify the range of significance based on 1000+ permutations.

We have redone the burden test with population-matched groups to confirm the reliability of this association (lines 435 - 446). In addition, we now acknowledge in the Caveats and limitation section that our observations could benefit from a permutation analysis (lines 695 - 697).

Page 17, bottom- Statements like these are overstating the correlation as the comparative analyses were not shown.

We agree and have edited the text to avoid potential overstatements.

This is good to include the role of female reproductive tract. Shouldn't the unbiased screen pull these out anyway? The authors did find some female GO terms enriched. What additional information or experiments would be needed to test the hypothesis of female compensation? The expectations for this should be made clearer.

Given the nature of these putative female compensatory mechanisms (primarily acting on the oviduct and lower uterus, as speculated in lines 586 – 601), it is currently impossible to functionally test them in gorillas. The continued development of in vitro systems mimicking the female reproductive tract may allow such studies in the future.

Page 18, middle- Pleiotropy is an important consideration and this paragraph discusses some valuable points. However, this is another section that could be improved by discussing the relaxed constraints in later spermatogenesis, which likely suggests that genes expressed in later stages are less pleiotropic and more testis- specific.

We agree and have added a brief discussion of this in lines 619 - 622: “It is also possible that the negative consequences of deleterious pleiotropy become less pronounced at later stages of spermatogenesis as meiotic and post-meiotically expressed genes are enriched for testis-specific functions (PMID: 36544022).”

Page 27, Bottom- The criteria for selection of genes to target here is interesting and disconnected from the claimed interpretation of the results. If you're targeting genes with reliable expression in Drosophila, it is not surprising that a percentage of them will lead to fertility loss. Shouldn't the background be a random set of testis-expressed genes? This test would show that relaxed constraint is a strong way to screen for fertility genes. Additionally, the authors previously showed that these genes were enriched in SC-rna in gorilla,- and likely other species. Suggesting that you identified genes 'lacking evidence' of a role in spermatogenesis in previous studies is misleading, when many of these genes are present in testis RNA datasets and enriched for sperm go terms. I would argue that genes found to be expressed in testis and spermatogenesis specific cell types, certainly have evidence of being involved in spermatogenesis.

We thank you for the helpful suggestion. We have generated a new background group composed of a random set of testis-expressed genes. More specifically, by looking at previously published Drosophila testis expression data (PMID: 30249207), we randomly selected 156 genes with TPM>1 (transcript per million) and determined the percentage of them with reported spermatogenic / male fertility defects in Drosophila. We observed that 18 (11.5%) had been previously demonstrated to be functionally required for male reproductive fitness. This percentage is slightly higher than what we had previously observed for a random selection of Drosophila genes (9.6% - an update, using the latest available data, to the 7.7% reported in the original version). Nevertheless, both figures are still well below the 27.6% hit rate we found for the Drosophila orthologs of the gorilla K<1 genes. We have added this new information to the manuscript (lines 380 - 386).

Regarding the potential correlation between expression and function in spermatogenesis, we and others have shown that the majority of the protein-coding genome is expressed during spermatogenesis in both vertebrate and invertebrate species (PMID: 39388236). Although the reasons for such widespread transcription in the male germ line are not entirely clear, it advises a cautious approach in terms of correlating expression with function. Indeed, our recent analysis of 920 genes reliably expressed in insect and mammalian spermatogenesis revealed that only 27.2% of them caused male reproductive impairment when individually silenced in the Drosophila testis (PMID: 39388236). Since genetic redundancy is a factor that needs to be taken into consideration when dealing with such a central biological process for the survival of a species, we take the more stringent approach of only considering a gene to be functionally involved in spermatogenesis if there is phenotypical evidence (from our RNAi assay or from previous publications) that its disruption is associated with spermatogenic impairment and/or abnormal fertility. We have added this clarification to the manuscript (lines 349 - 363).

Page 17 "Our data ... suggests that gorillas may be at the lowest limit of male reproductive function that can be maintained by natural selection (at least in mammals or vertebrates)." I realize this is the speculation section, but this is a massive overstatement. There is absolutely nothing in your data or results that support this statement, nor is this supported by the extensive comparative reproductive data in mammals. For example, there are many mammalian systems that show lower metrics of reproductive function than gorillas. For example, the sperm abnormality indices in Box 1F are nowhere near as severe as found in many species that still somehow manage to reproduce.

We agree and have edited the text to avoid potential overstatements (see above).

**Reviewer #3 (Recommendations for The Authors):**

(1) More discussion is needed as to whether their results could be explained by a reduction in effective population size in gorillas.

Thank you for raising this important point. As you know, reduced effective population size can lead to an increased load of deleterious mutations/relaxed selection intensity. However, we do not believe that it substantially affects our observations. Indeed, relatively few genes have K<1 and those are enriched in sperm biology. Given that a reduced effective population size will plausibly increase the load of deleterious mutations and relaxed selection across many genes, it is unlikely that such a broad phenomenon would result in a specific enrichment in genes related to male reproductive biology. We have added this reasoning to the Caveats and limitations section (lines 675 - 682).

(2) Properly controlled genetic association testing when performing a burden test is essential, and methods that allow for some variants to be associated with increased fertility should be considered. Rare variants are much more likely to show population-specific differences, and selecting humans from two potentially very different cohorts and sample sizes can easily lead to confounding. I suggest performing a principal component analysis to ascertain the degree of genetic differentiation between these cohorts, and use this to guide the selection of a subset of the control cohort as well.

We agree and have replicated this analysis using only individuals of European descent; our conclusions have not changed but the P-values have become lower (lines 435 - 446).

(3) Citations should also be included in Table 1, for each relevant phenotype. You may also want to consider a more general comparison of p-values and effect sizes of genome-wide association studies for human male infertility to test for an enrichment in/nearby genes showing relaxed selection along the gorilla lineage. In other words, do the relaxed genes in the gorilla lineage have an enrichment of small p-values for being associated with male infertility.

Citations have been included in Table 1, as suggested, and the table has been updated to include the latest reported phenotypes.
